# Thrombin Determination Using Graphene Oxide Sensors with Co-Assisted Amplification

**DOI:** 10.3390/mi13091435

**Published:** 2022-08-31

**Authors:** Lei Liu, Qin Li, Haixia Shi, Li Gao

**Affiliations:** 1 Department of Kidney Transplantation, The Second Xiangya Hospital of Central South University, Changsha 410011, China; 2 School of Life Sciences, Jiangsu University, Zhenjiang 212013, China; 3 Physical Education Department, Jiangsu University, Zhenjiang 212013, China

**Keywords:** graphene oxide (GO), polyethylene glycol (PEG), aptamer, high sensitivity, thrombin detection

## Abstract

Graphene oxide (GO) is widely used in sensors. The detection of proteins based on bare GO has been developed; however, the detection sensitivity needs to be improved. In this paper, a novel GO-DNA sensor for thrombin detection was developed using an aptamer linked to the surface of GO. Polyethylene glycol (PEG) was further used to prevent thrombin from nonspecific adsorption and to improve the sensitivity of the sensor for detection of thrombin. In order to improve the limit of detection for thrombin, we developed a GO and RecJf exonuclease co-assisted signal amplification strategy, and a detection limit of 24.35 fM for thrombin was achieved using this strategy. The results show that it is a promising method in analytical applications.

## 1. Introduction

Thrombin is a serine protease in the blood that participates in physiological and pathological reactions such as inflammation, wound repair, blood coagulation, and platelet activation [1,2]. Variations in the concentration levels of thrombin in blood can cause abnormal coagulation function [3]. In addition, thrombin is closely related to the development of many diseases and is used as a disease marker [4,5,6]. Therefore, a high-sensitivity method for detection of thrombin is important in the early stage of clinical diagnosis. Some methods have been widely applied for thrombin detection, including electrochemistry [7,8], surface plasmon resonance (SPR) [9], colorimetry [10], immunosorbent assay (ELISA) [11], and amperometry [12]. However, some detection approaches lack high sensitivity or selectivity, and some methods are complicated. Therefore, there is a need to develop a simple and highly sensitive method. Aptamers are flexible, repeatable, easy to fix, and regenerate with no differences between batches, and they have been widely used in the biosensor field [13]. Recently, studies have applied aptamer-based biosensors for the detection of thrombin due to their good sensitivity and selectivity, high accuracy, fast response, and low cost [14,15]; however, detection sensitivity needs to be improved.

Graphene oxide (GO) has two characteristics: First, it is a high-efficiency quenching agent. The fluorescent group used in the experiment was quenched near the surface of GO. Second, it can interact with DNA through π-π bonds and hydrogen bonding [16]. Therefore, it has strong adsorption to single-stranded DNA and has a high signal-to-noise ratio. When a target molecule exists, adsorption is relieved [17]. The surface of GO is rich in hydrophilic groups such as alkyl groups, epoxy groups, and carboxyl groups, which can be evenly dispersed in water. Therefore, GO-based sensors have attracted special attention, due to their short assay time, relatively low cost, and no requirement for skillful technicians [18,19,20,21,22,23]. The carboxyl groups on the surface of GO conjugate with the amino-modified aptamer, and therefore, the aptamer fixes on the surface of GO. Other molecules can not be conjugated. The detection accuracy is improved and the appearance of false positive signals is avoided [18,24]. A target protein (thrombin) can still be adsorbed on the surface of GO. In order to solve these problems, a resisting nonspecific displacement probe covalently linked to GO and PEG has been further used to prevent protein (thrombin) from nonspecific binding to the GO. In this study, a GO and exonuclease co-assisted signal amplification strategy was further developed in order to improve the protein detection limit.

## 2. Materials and Methods

### 2.1. Chemicals and Materials

GO was synthesized from natural graphene powder using the modified Hummers method [24]. The human α-thrombin with purity more than 95% was obtained from Haematologic Technologies Inc. (Essex Junction, VT, USA). The exonuclease was purchased from New England Biolabs (Beijing) Ltd. (Beijing, China). The PEG and other proteins were purchased from Sigma-Aldrich Chemical Co., Ltd. (Shanghai, China).

As shown in Table 1, the underlined sequences could be a hairpin structure. The aptamer was purchased from Sangon Biotechnology Co., Ltd. (Shanghai, China) with HPLC purification [14]. The underlined sequences could be easily recognized by RecJf exonuclease (Exo). The PBS buffer was purchased from Shanghai Double Helix Biotechnology Co., Ltd. (Shanghai, China). The human blood serum samples were obtained from Affiliated Hospital of Jiangsu University. 

### 2.2. Sensor Preparation

The prepared GO powder was dispersed in ultrapure water, and then sonicated for 0.5 h (1000 W). A uniform GO dispersion was obtained. A solution was prepared containing a mixture of 50 mM NHS and 200 mM EDC in ultrapure water and GO solution according to the volume ratio of 1:1:2. The solution was centrifuged at 1000 rpm for 20 min to activate GO. In order to immobilize the DNA on the surface of GO, 20 μg/mL of activated GO was added to 200 μL of 10 nM DNA. The reaction time was 12 h at 4 °C [25]. Then, it was centrifuged at 1000 rpm for 20 min to remove the immobilized DNA sequence.

### 2.3. Sensitivity for Thrombin Detection

The 1 mL PBS contained 20 μg/mL of GO and 10 nM of DNA. Then, different concentrations of thrombin were added and incubated for 30 min for the detection. RecJf exonuclease digestion was carried out at 37 °C in a buffer including 10 mM Tris hydrochloride (Tris-HCl), 50 mM NaCl, 10 mM MgCl_2_, and 1 mM dithiothreitol (DTT, pH 7.9). The fluorescence intensity was detected using a Synergy™ H_4_ Hybrid Multi-Mode Microplate Reader (BioTek Inc., Winooski, VT, USA). The emission spectra were recorded in the wavelength of 510–600 nm with excitation at 480 nm. The curves were made using the fluorescence intensity at 520 nm. The data obtained in the experiment were processed using Origin 8.0. The value of F/F_0_-1 is the ratio of fluorescence intensity. F and F_0_ are the fluorescence intensities with thrombin and without thrombin, respectively.

### 2.4. Selectivity for Thrombin Detection

Other proteins (lysozyme, IgG, and BSA) were added with 0.1 nM into 10 nM aptamer and 20 μg/mL GO under the same conditions as thrombin, and incubated at room temperature for 30 min for detection of fluorescence intensity.

## 3. Results and Discussion

### 3.1. Design Strategy for Thrombin Detection

**Scheme 1:**
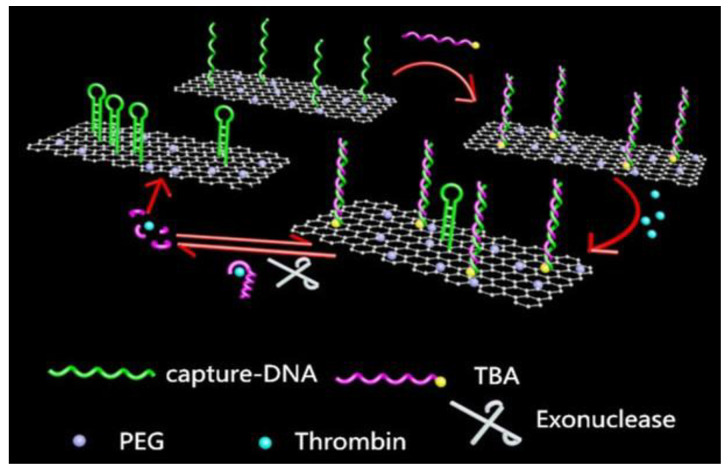
The strategy for thrombin detection using the exonuclease co-assisted amplification strategy.

Scheme 1 shows that the strategy for the grahene oxide (GO)-aptamer sensor to detect thrombin. The amino-modified capture DNA was immobilized to the activated GO surface using the covalent bonds with the COOH group on the surface of GO. PEG was applied on the GO surface to prevent the nonspecific adsorption of thrombin binding aptamer (TBA) and protein. Then, TBA modified with FAM (carboxyfluorescein) was added. Capture DNA was partially complementary to TBA. Therefore, FAM labeled on the 3′ end of the aptamer was quenched by GO using fluorescence resonance energy transfer (FRET) between GO and FAM. When thrombin was captured by the aptamer, the structure of the TBA–thrombin complex changed and separated from the GO surface, and the fluorescence intensity recovered. RecJf Exo degraded DNA in the direction 5′→3′. Capture DNA formed a hairpin-shaped structure on the surface of GO and was not recognized by RecJf exonuclease. The RecJf exonuclease in the solution recognized the single-stranded TBA in the TBA–thrombin complex and hydrolyzed it to release thrombin into the solution for recycling. Thrombin continued to bind to TBA on the GO surface, which enhanced the fluorescence intensity. PEG was reported as a blocking agent to prevent the absorbability of nonspecific binding materials. It is a nonionic surfactant that may strongly interact with GO through its hydrocarbon lipophilic group. This interfered with the formation of aptamers or protein/GO complexes [26]. It was used to prevent the protein from nonspecific binding to improve the detection limit in the following detection strategy.

### 3.2. Sensitivity for Thrombin Detection Using a Covalent Linking Aptamer with PEG-Based Sensor

**Figure 1:**
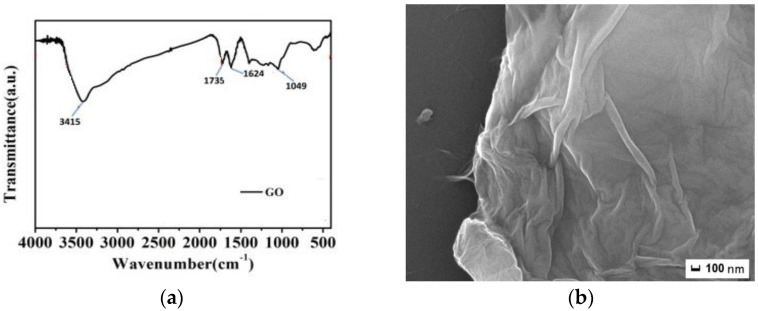
FT−IR spectrum (**a**) and scanning electron micrograph image (SEM) (**b**) of GO.

**Figure 2:**
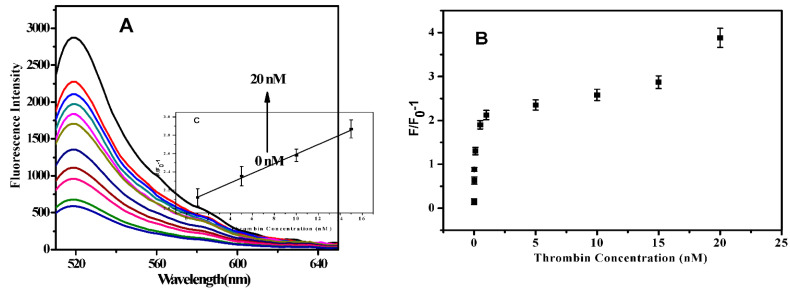
(**A**) The fluorescence intensity of the GO-DNA sensor by covalent linking in the presence of different concentrations of thrombin (0.00025, 0.005, 0.01, 0.1, 0.5,1, 5, 10, 15, and 20 nM); (**B**) the values of F/F_0_-1 for the assay with the concentrations of thrombin; (**C**) the values of F/F_0_-1 for detection with the concentrations of thrombin. The number of samples was 5.

As shown in Figure 1a, several characteristic peaks of GO were observed in the FT-IR spectrum including the peaks at 3386, 1718, 1618, and 1050 cm^−1^, due to O-H stretching vibration, C=O stretching vibration, C-OH stretching vibration, and C-O stretching vibration, respectively. This showed that GO was successfully synthesized [27]. Figure 1b shows the SEM of GO. The fluorescence intensity of different concentrations of FAM-DNA with the addition of various concentrations of GO using a covalent linking aptamer are shown in Figure S1. Note, 90% of the fluorescence intensity was quenched when GO was 5 μg/mL. A GO concentration lower than 5 μg/mL was not enough, and a GO concentration higher than 5 μg/mL was redundant. Therefore, 5 μg/mL of GO was chosen. When thrombin was added, the change of fluorescence intensity was the highest at 10 nM. Therefore, 10 nM DNA was selected. The fluorescence intensity of the GO-DNA sensor by covalent linking with the change of time after added thrombin is shown in Figure S2. The release of aptamer from GO required almost 30 min. Therefore, 30 min was chosen as the incubation time. As shown in Figure 2A, the detection signal became stronger with thrombin concentrations increasing from 0.0025 to 15 nM. There is a linear relationship between the thrombin concentrations and the values of F/F_0_-1 (R^2^ = 0.99), as shown in Figure 2B. The limit of detection (LOD) for thrombin was calculated as 0.11 pM based on the 3σ/slope over the range 1–15 nM (Figure 2C).

**Figure 3:**
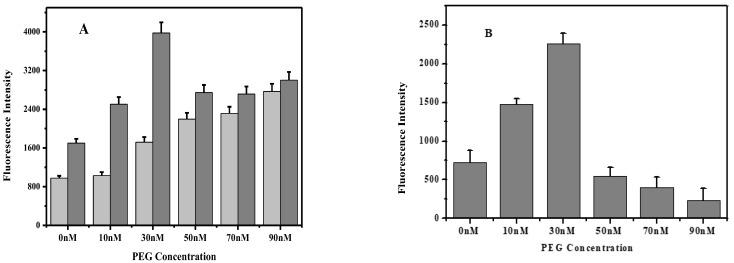
(**A**) Effect of PEG concentrations on the GO-DNA sensor for fluorescence intensity (aptamer adsorpted on the surface of GO without (gray bar) and with (dark grey bar) thrombin); (**B**) effect of PEG concentrations on the GO-DNA sensor for change of fluorescence intensity. The number of samples was 5.

**Figure 4:**
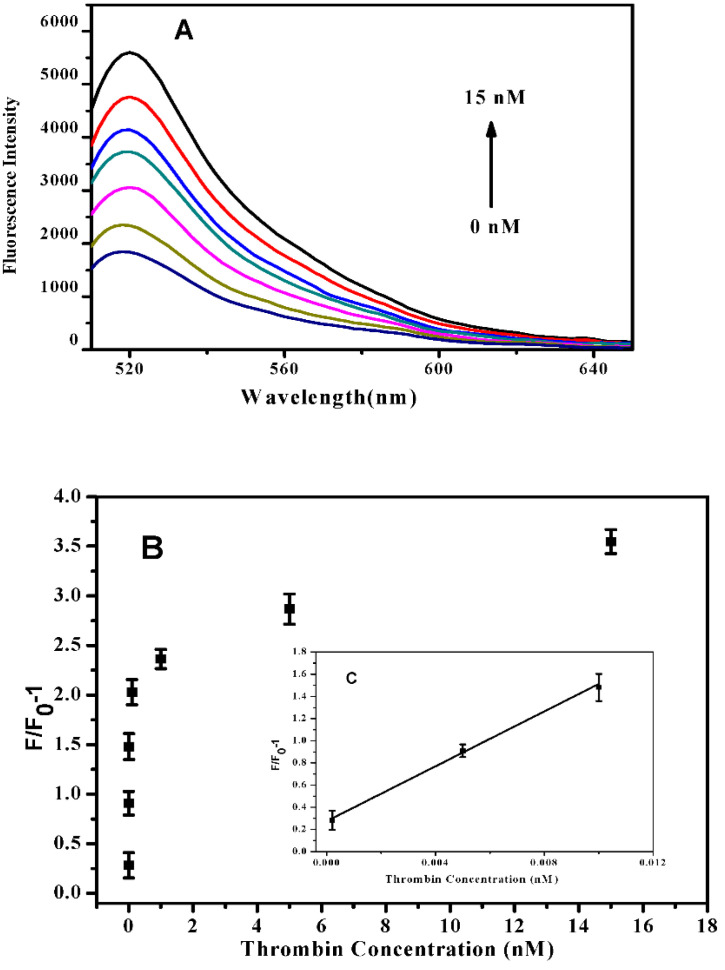
(**A**) The detection of different concentrations of thrombin from bottom to top (0.0002, 0.05, 0.01,1, 5, and 15 nM) using the GO-DNA sensor; (**B**) the values of F/F_0_-1 for the assay with the concentrations of thrombin; (**C**) the values of F/F_0_-1 for the detection of thrombin. The number of samples was 5.

As illustrated in Figure 3A, the effect of PEG concentration on the GO-DNA sensor for fluorescence intensity is shown when the aptamer is adsorpted on the surface of GO without (gray bar) and with (dark grey bar) thrombin. Figure 3B shows the values of F/F_0_-1. After adding PEG, the increased fluorescence intensity is obvious with 30 nM PEG. As shown in Figure 3B, the fluorescence intensity of the solution increases with the addition of PEG up to 90 nM because PEG can improve the fluorescence intensity. As shown in Figure 4A, the curve shows that the fluorescence intensity increases as the concentration of thrombin increases. The inset in Figure 4B reveals a linear correlation (R^2^ = 0.98) between the value of F/F_0_-1 and the concentration of thrombin over the range 0.0002–15 nM. The detection limit was improved to 0.034 pM based on 3σ/slope lower than the 0.11 pM without PEG.

### 3.3. Sensitivity of the Detection for Thrombin Using a Covalent Linking Aptamer with Exonuclease Co-Assisted Amplification Strategy

**Figure 5:**
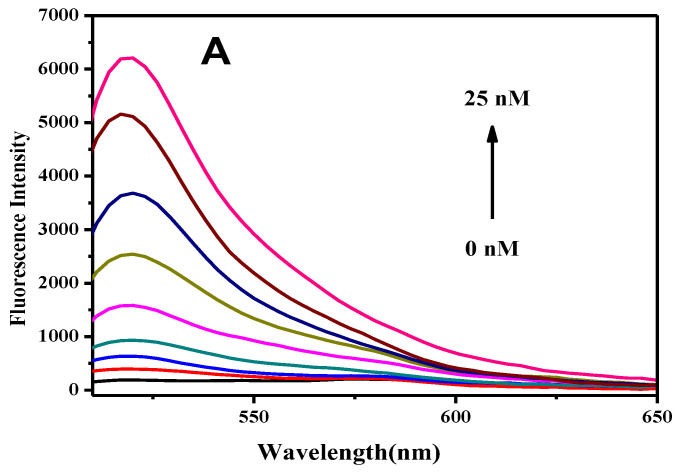

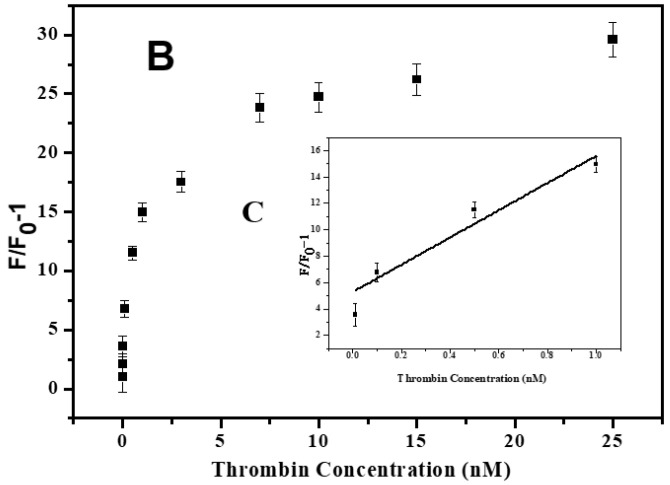
(**A**) The detection of different concentrations of thrombin (0.000125, 0.001, 0.01, 0.1, 0.5, 3, 7, 20, and 25 nM were seperately for color lines from bottom to top) using the GO-DNA sensor; (**B**) and the inset (**C**) the values of F/F_0_-1 for the assay with the concentrations of thrombin. The number of samples was 5.

As shown in Figure S3, the red curve represents the increased fluorescence intensity produced by thrombin without exonuclease and the blue curve represents the fluorescence signal under the reaction of exonuclease. This highlights the effect of exonuclease. As shown in Figure S4, 90% of the fluorescence intensity of DNA with FAM was quenched and trended to a minimum value at 20 μg/mL GO. Therefore, 20 μg/mL of GO was used for the following experiments. As shown in Figure S5, the fluorescence intensity increased rapidly in the presence of 0.03 U μL^−1^ RecJf exonuclease at 37 °C in buffer (50 mM NaCl, 1 mM DTT, 10 mM MgCl_2_, and 10 mM Tris–HCl with pH 7.9) [28]. Thirty minutes was chosen as the incubation time due to the complete digestion of DNA in 30 min for the next recycle. In addition, the detected fluorescence intensity of thrombin in water was higher than in 0.01 M PBS in the presence of RecJf exonuclease, as shown in Figure S6. Therefore, water was a dissolved buffer in the detection of thrombin. Figure 5A illustrates the fluorescence intensity of the GO-DNA sensor with different thrombin concentrations. Figure 5B shows the calibration curve of fluorescence intensity and a linear correlation (R^2^ = 0.97) between the values of F/F_0_-1 and the concentrations of thrombin over the range 0.000125–25 nM. LOD was improved to 24.35 fM based on the 3σ/slope (Figure 5B). 

As shown in Table 2, LOD is lower than that obtained from other assays. In addition to LOD, this method is simple and inexpensive. Therefore, it is a promising method for thrombin detection.

### 3.4. Selectivity of the Thrombin Detection with the Exonuclease Co-Assisted Amplification Strategy

**Figure 6:**
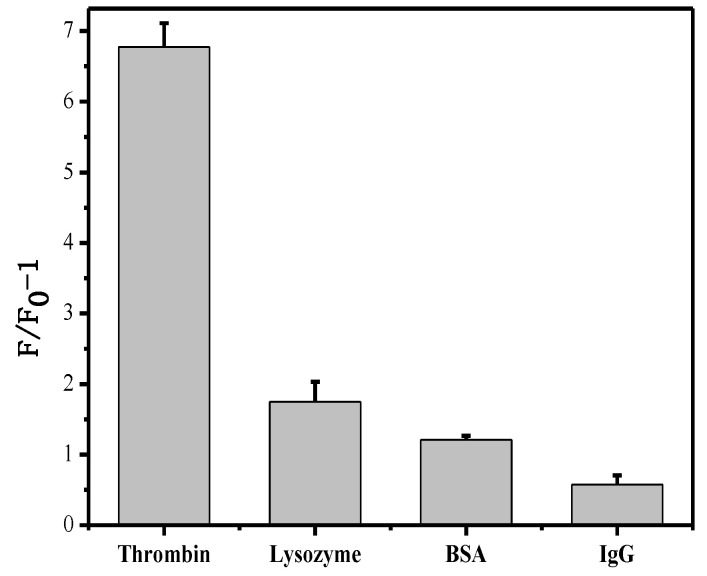
The fluorescence intensity of aptamer (10 nM)-GO (20 μg/mL) in the presence of other proteins (BSA, Ig G, and lysozyme, the concentration was 0.001 nM for every protein). The number of samples was 5.

To evaluate the specificity of the GO-based aptamer for thrombin, the influences of some relevant biological species including IgG, BSA, and lysozyme were detected (Figure 6) as negative controls, each at an identical concentration of 0.001 nM. Thrombin resulted in significant enhancement as compared with other proteins because other proteins could not specifically bind to the aptamer. This shows the sensor has good selectivity for thrombin detection.

### 3.5. The Application of Exonuclease Co-Assisted Amplification Detection

In order to further study the potential application of the biosensor, thrombin was detected in real samples in order to make a better reliability evaluation of the sensor application analysis. The Affiliated Hospital of Jiangsu University provided human blood serum samples. Each sample was carried out three times. Table 3 shows the detection results. The recovery (between 96.56% and 102.62%) and relative standard deviation (RSD) (between 7.22% and 10.41%) were acceptable. These results show that the method is promising in real samples.

## 4. Conclusions

Here, we developed an immobilized aptamer on the surface of graphene oxide and exonuclease co-assisted amplification for thrombin detection. In this study, this sensor was resistant to false positive signals and nonspecific probe displacement. The adsorption of thrombin on the surface of GO was prevented by using PEG. Thrombin was released by using RecJf exonuclease to digest the single-stranded aptamer, which could then be detected for the next recycle. The detection limit was 24.35 fM. The detection sensitivity was improved in this method. This method has great potential for early detection of diseases.

## Figures and Tables

**Table 1:** DNA sequences for this experiment.

Thrombin binding aptamer (TBA)	5′-AAAAGTCCGTG GTAGGGCA GGTTGGGGTGACT-FAM-3′
The complementary sequence of thrombin aptamer	5′-NH_2_–AGTCACCCCAACCTGCC CTACCACGGACT-3′

**Table 2:** Different methods for thrombin detection based on aptamer sensors.

Detection Technique	Linear Range	Detection Limit	Reference
Fluorescence	From 0.05 pM to 200 pM	0.05 pM	[29]
Fluorescence	From 20 pM to 200 pM	9.2 pM	[30]
Surface plasmon resonance (SPR)	From 0.1 nM to 75 nM	0.1 nM	[31]
Electrochemistry (differential pulse voltammetry (DPV)	From 1pM to 30 nM	0.32 pM	[32]
Electrochemistry (differential pulse voltammetry (DPV)	From 1 pM to 10 nM	0.64 pM	[33]
Flow strip biosensor (LFB)	From 6.4 pM to 500 nM	4.9 pM	[34]
Colorimetric method	From 1.3 nM to133 nM	0.61 nM	[35]
Fluorescence	From 0.000125 nM to 25 nM	24.35 fM	Present work

**Table 3:** Detection of thrombin in human blood serum (the number of samples was 5).

Serum Sample	Concentration of Thrombin Added (nM)	Concentration Obtained with Aptasensor (nM)	Recovery/%	RSD/%
1	0.000125	0.000128	102.62	10.41
2	0.001	0.0009656	96.56	9.37
3	0.1	0.009983	99.83	7.22

## Data Availability

Data available on request due to restrictions eg privacy or ethical. The data presented in this study are available on request from the corresponding author.

## Supplementary Materials

The following supporting information can be downloaded at: https://www.mdpi.com/article/10.3390/mi13091435/s1, Figure S1. (A)The fluorescence intensity of different concentrations of FAM-DNA in the addition of various concentrations of GO. (B) Effect of different concentrations of DNA for thrombin detection; Figure S2. The fluorescence intensity of GO-DNA sensor by covalent linking with the change of time after added thrombin (1 nM); Figure S3. The fluorescence intensity of TBA in blank (black), with thrombin (red), and with 0.03U/μL exonuclease (blue); Figure S4. (A)The fluorescence intensity of TBA in the presence of various concentrations of GO (2, 6, 10, 12, 15, 20, 25 μg/mL); (B) The Influence of GO concentration for the detection of thrombin; Figure S5. The fluorescence intensity of TBA changing with time (Black:10 nM DNA-20 μg/ml GO, Red: 10 nM DNA-20 μg/ml GO- (1nM) Thrombin-Exonulase (0.03 U/μL); Figure S6. The Influence of buffer environment for the detection of thrombin.
