# Supplementary material for: Thrombin Determination Using Graphene Oxide Sensors with Co-Assisted Amplification
Source: Micromachines (Basel). 2022 Aug 31;13(9):1435. doi: 10.3390/mi13091435 (PMC9502102; doi:10.3390/mi13091435)
Supplement: Supplementary file 1 [file micromachines-13-01435-s001.zip › micromachines-1850084-supplementary.pdf]

Thrombin Determination Using Graphene Oxide Sensors with Co-assisted Amplification

Lei Liu[a] , Qin Li[b], Haixia Shi[c]* , Li Gao[b]*

[a]Department of Kidney Transplantation, The Second Xiangya Hospital of Central South University, Changsha, 410011, P.R.China

[b] School of Life Sciences, Jiangsu University, Zhenjiang 212013, P. R. China;

[c]P.E.department of Jiangsu University, Zhenjiang 212013, P. R. China

Correspondence: gaoli@ujs.edu.cn

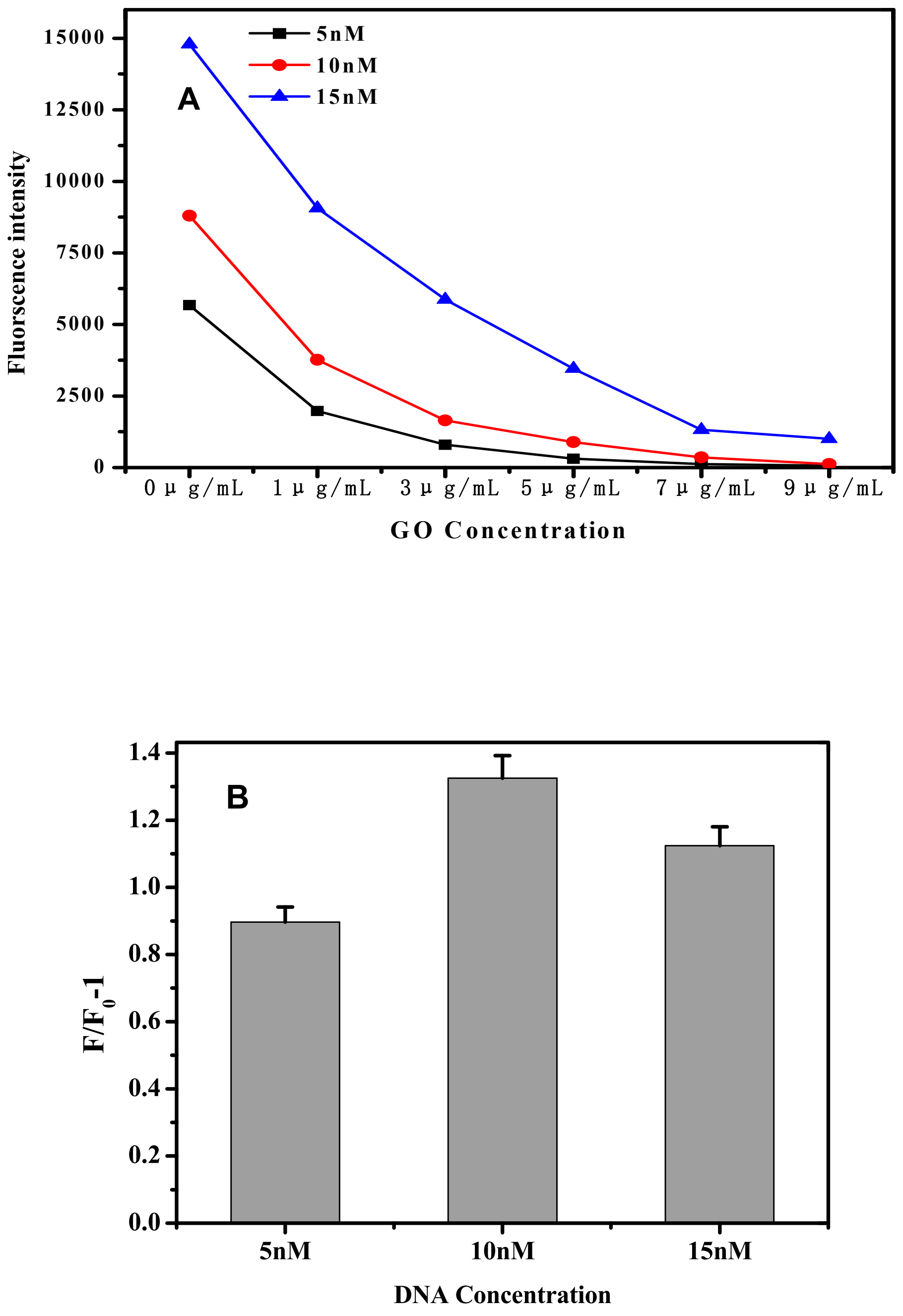

Figure S1. (**A**)The fluorescence intensity of different concentrations of FAM-DNA in the addition of various concentrations of GO. (**B**) Effect of different concentrations of DNA for thrombin detection.

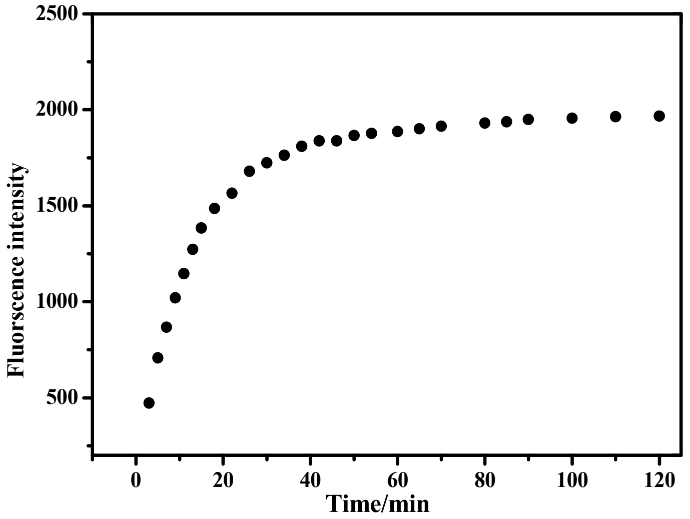

Figure S2. The fluorescence intensity of GO-DNA sensor by covalent linking with the change of time after added thrombin (1 nM)

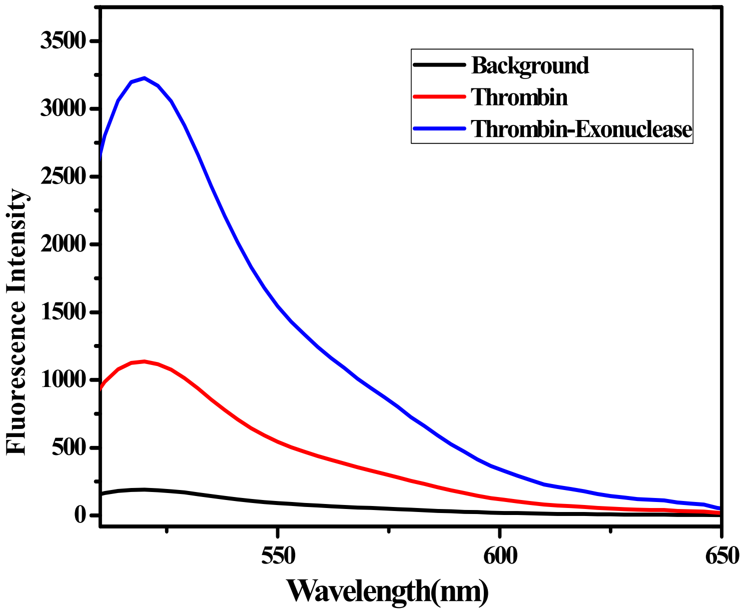

Figure S3. The fluorescence intensity of TBA in blank (black), with thrombin (red), and with 0.03U/μL exonuclease (blue).

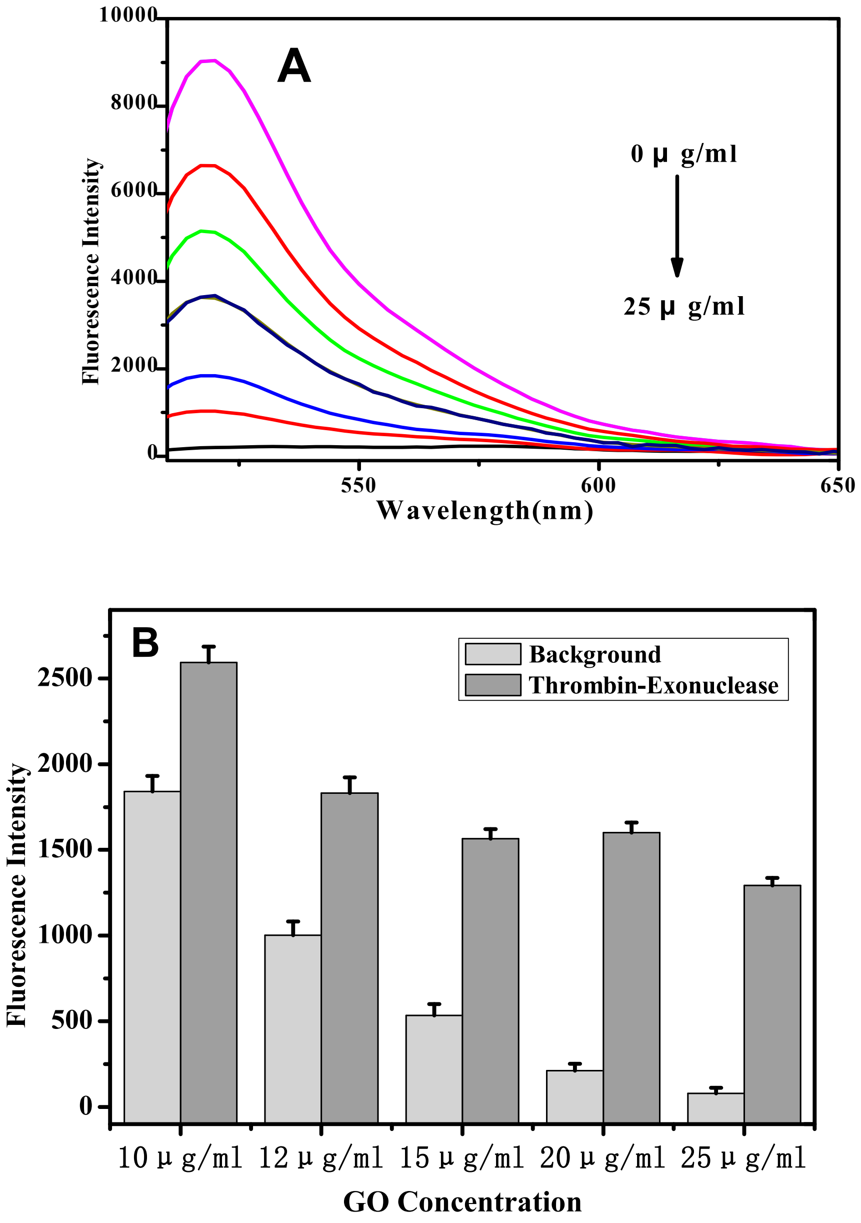

Figure S4. (**A**)The fluorescence intensity of TBA in the presence of various concentrations of GO (2, 6, 10, 12, 15, 20, 25 μg/mL); (**B**) The Influence of GO concentration for the detection of thrombin.

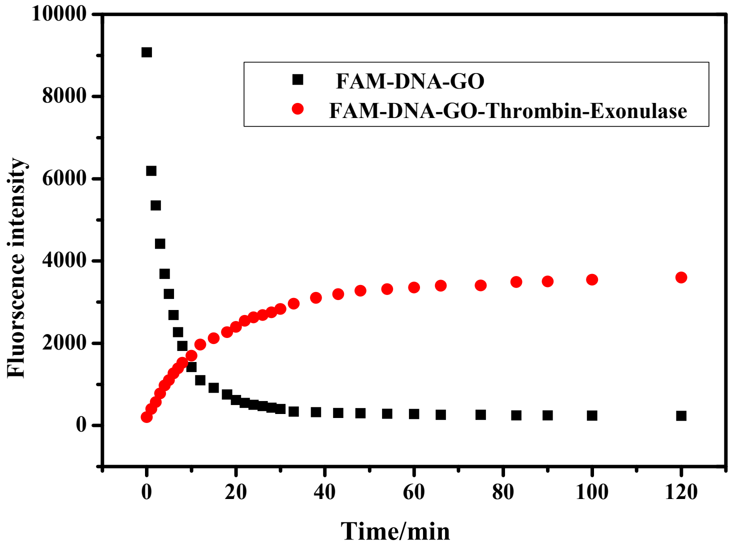

Figure S5. The fluorescence intensity of TBA changing with time (Black:10 nM DNA-20 μg/ml GO，Red: 10 nM DNA-20 μg/ml GO- (1nM) Thrombin-Exonulase (0.03 U/μL)

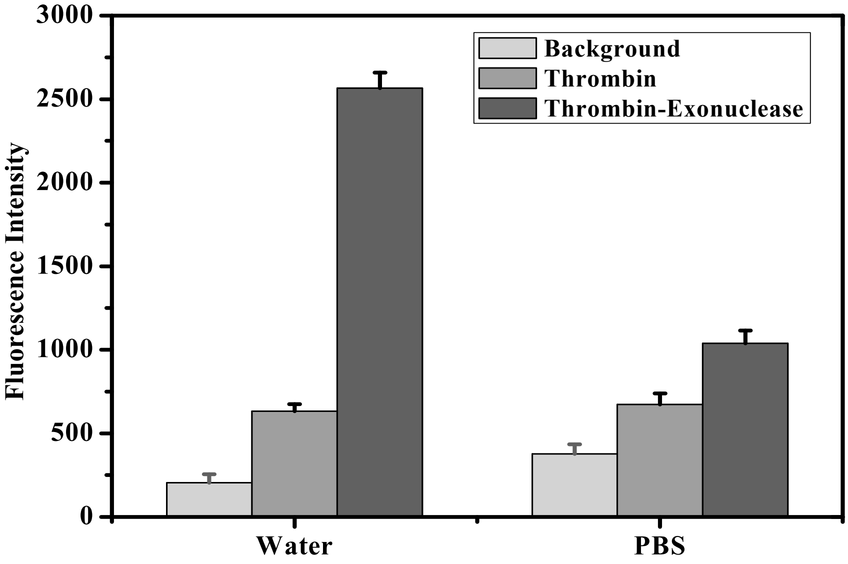

Figure S6. The Influence of buffer environment for the detection of thrombin
